# Transient Receptor Potential Channel Canonical Type 3 Deficiency Antagonizes Myofibroblast Transdifferentiation In Vivo

**DOI:** 10.1155/2020/1202189

**Published:** 2020-03-05

**Authors:** Weijie Xia, Qianran Wang, Yuangang Lu, Yingru Hu, Xingcun Zhang, Junbo Zhang, Dongfang Liu, Jinlin Song, Zhiming Zhu, Daoyan Liu, Hengshu Zhang

**Affiliations:** ^1^Department of Burn & Plastic Surgery, The First Affiliated Hospital of Chongqing Medical University, Chongqing 400016, China; ^2^Department of Endocrinology, The Second Affiliated Hospital, Chongqing Medical University, Chongqing 400010, China; ^3^Department of Plastic & Cosmetic Surgery, Research Institute of Surgery, Daping Hospital, Third Military Medical University, Chongqing 400042, China; ^4^Department of Hypertension and Endocrinology, Center for Hypertension and Metabolic Diseases, Daping Hospital, Third Military Medical University, Chongqing Institute of Hypertension, Chongqing 400042, China; ^5^School of Dental Medicine, Chongqing Medical University, Chongqing 400016, China

## Abstract

**Objective:**

Myofibroblast transformation has been shown to be associated with the reactive oxygen species- (ROS-) producing enzyme NADPH oxidase (Nox4). Inhibition of transient receptor potential channel canonical type 3 (TRPC3) attenuates mitochondrial calcium handling and ROS production in the vasculature of hypertensive rats. However, it remains elusive whether TRPC3 regulates mitochondrial calcium and ROS production and participates in myofibroblast transdifferentiation during wound healing.

**Methods and Results:**

In this study, we demonstrated that activation of TRPC3 by transforming growth factor *β* (TGF*β* (TGF*α*SMA). Inhibition of TRPC3 with its specific inhibitor, Pyr3, significantly decreased TGF*β* (TGF*α*SMA). Inhibition of TRPC3 with its specific inhibitor, Pyr3, significantly decreased TGF*β* (TGF*β* (TGF*Trpc3^−/−^* mice exhibited significantly attenuated myofibroblast transdifferentiation, as demonstrated by decreased *α*SMA). Inhibition of TRPC3 with its specific inhibitor, Pyr3, significantly decreased TGF*β* (TGF*β* (TGF*Trpc3^−/−^* mice exhibited significantly attenuated myofibroblast transdifferentiation, as demonstrated by decreased *Trpc3^+/+^* mice. In addition, *Trpc3^−/−^* mice exhibited significantly attenuated myofibroblast transdifferentiation, as demonstrated by decreased

**Conclusions:**

Our data indicate that TGF*β*1-mediated activation of TRPC3 enhances mitochondrial calcium and ROS production, which promotes myofibroblast transdifferentiation and HTS formation. Inhibition of the TRPC3-mediated Nox4/pSmad2/3 pathway may be a useful strategy to limit HTS formation after injury.*β* (TGF

## 1. Introduction

Hypertrophic scars (HTSs) often lead to dysfunction, damaged appearance, and psychological discomfort, and the current clinical treatments are not optimal [1]. Myofibroblasts are overabundant in HTS tissue after burn injury [2]. Myofibroblast proliferation is initiated by the formation of granulation tissue and the recruitment of activating myofibroblasts, which play central roles in extracellular matrix (ECM) deposition, reepithelialization, and eventual wound closure. The major components of HTS tissue are collagen-1 (Col1a1) and fibronectin, which mediate ECM remodelling, and *α*-smooth muscle actin- (*α*SMA) expressing myofibroblasts, which provide contractile strength [3]. Myofibroblast transdifferentiation associated with HTS formation plays a central role during wound healing. However, the molecular mechanisms remain unclear.

Transforming growth factor *β* (TGF*β*) is a cytokine that may promote myofibroblast transdifferentiation during acute tissue injury. TGF*β* plays important roles in regulating proliferation and differentiation as well as in tissue fibrosis [4, 5]. TGF*β* induces de novo synthesis of *α*SMA fibres that enhance contractility and increases the protein expression of the ECM proteins collagen and fibronectin after injury. Mammals express the TGF*β* isoforms TGF*β*1-TGF*β*3. TGF*β*1 regulates the proliferation of keratinocytes and dermal fibroblasts, including in chronic ulcers [6]. Notably, Smad proteins are among the most important intracellular signal transduction proteins downstream of the TGF*β* superfamily [7]. TGF*β*1 acts through a heterodimeric receptor at the plasma membrane that phosphorylates the transcription factors Smad2 and Smad3 [8]. Disruption of the TGF*β*1/Smad3 signalling pathway due to loss of Smad3 confers resistance to tissue fibrosis in the skin, kidneys, lungs, and liver [9, 10].

Mitochondrial Ca^2+^ uptake is critical for the regulation of numerous cellular processes and of energy metabolism, but Ca^2+^ overload in the mitochondrial matrix impairs mitochondrial function and leads to reactive oxygen species (ROS) generation [11]. Mitochondrial pyruvate dehydrogenase (PDH) and several electron transport complexes are associated with changes in mitochondrial Ca^2+^ homeostasis [12]. NADPH oxidase (Nox) 4 utilizes electrons from NADPH to generate superoxides, and suppression of Nox4 has been found to decrease myofibroblast formation and fibrosis in several tissue injury models [13]. Recently, TGF*β*-induced myofibroblast transformation has been shown to be associated with the ROS-producing enzyme Nox4 [1]. However, the mechanisms of mitochondrial Ca^2+^ overload through ROS and the role of the Nox4/Smad3 signalling pathway in regulating myofibroblast transformation remain unknown.

Transient receptor potential (TRP) channels play important regulatory roles in cellular Ca^2+^ homeostasis, growth, migration, and inflammatory mechanisms [14, 15]. TRP channels are involved in dermatological disorders [16], but the function of TRP channels in myofibroblast transdifferentiation is poorly understood. TRPC6 is known to activate the Ca^2+^-responsive protein phosphatase calcineurin to induce myofibroblast transdifferentiation and dermal wound healing [17], and TRPA1 promotes cardiac myofibroblast transdifferentiation after myocardial infarction injury [18]. Our previous studies have also revealed that enhancement of TRPC3 is associated with increased migration of monocytes [14] and with elevated mitochondrial Ca^2+^ uptake and ROS generation in the vasculature in hypertension [19]. However, little is known about whether TGF*β*1 can regulate mitochondrial Ca^2+^ and ROS production in the mitochondrial respiratory chain and elevate myofibroblast transdifferentiation by targeting TRPC3. Therefore, we hypothesized that TGF*β*1 enhances TRPC3-mediated mitochondrial Ca^2+^ uptake and ROS production, ultimately promoting myofibroblast transdifferentiation during wound healing and increasing HTS formation.

## 2. Materials and Methods

### 2.1. Tissue Samples and Cell Culture

Human HTS tissues obtained through surgical excision (*n* = 12, taken from eight women and four men with an age range of 23-55 years) were used for the experiments in this study. Nine HTS tissue samples from the face, 3 HTS tissue samples from the neck area, and some samples of corresponding adjacent normal skin tissue (*n* = 6) from the face (*n* = 3) and neck area (*n* = 3) were obtained during scar surgical excision at the Department of Plastic & Cosmetic Surgery, Daping Hospital, Army Military Medical University. This study was approved by the Ethics Committee of Daping Hospital, Army Military Medical University. All participants gave written informed consent.

Primary human fibroblasts were cultured in Dulbecco's modified Eagle's medium (DMEM) containing 10% foetal bovine serum (FBS) and antibiotics until they became confluent. Dermal fibroblasts from *Trpc3^+/+^* and *Trpc3^−/−^* were established as described previously [17]. The cells were cultured in DMEM (Gibco, China) supplemented with 10% FBS (HyClone, USA) containing 1% penicillin-streptomycin and were incubated in a 5% CO_2_ atmosphere at 37°C.

### 2.2. Animal Care and Open Wound Creation

*Trpc3^−/−^* mice and their *Trpc3^+/+^* littermates were obtained as gifts from Dr. Birnbaumer (Laboratory of Neurobiology, National Institute of Environmental Health Sciences, National Institutes of Health (NIH), Research Triangle Park, USA). The homozygotes, heterozygotes, and WT littermates were identified according to previously described methods [19]. Eight-week-old male *Trpc3^−/−^* mice (*n* = 6) and *Trpc3^+/+^* mice (*n* = 6) were maintained at a controlled temperature (21°C to 23°C) under a 12/12-hour light-dark cycle and with free access to food and water. All animal experimental procedures were approved by the Institutional Animal Care and Research Advisory Committee of the Army Military Medical University [15].

Open wounds were created on the backs of the mice (*Trpc3^−/−^* mice, *n* = 6; *Trpc3^+/+^* mice, *n* = 6). Each mouse was anaesthetized with pentobarbital (Matrx VIP 3000, Isoflurane Vaporizer, USA), and all limbs were extended evenly until the back skin became relaxed and symmetric. The back was sterilized using iodine and 70% EtOH, and an 8 mm circular excision line was drawn. The skin, including the panniculus carnosus, was carefully excised just above the myofascial layer with scissors. The wounds were washed using sterile 0.9% NaCl saline and sterile gauze dressings. The wound size was measured at 0, 3, 6, 9, 12, and 15 days after the wounds were created. The wound dressings were carefully removed with 0.9% NaCl saline, and care was taken not to change the wound size or shape. A standard ruler was used as a reference, and photographs of the wounds were taken with a digital camera (D80, Nikon, Tokyo, Japan). The wound areas were calculated using ImageJ software (public software, NIH).

### 2.3. Intracellular and Mitochondrial Ca^2+^ Measurement

The concentrations of cytosolic Ca^2+^ ([Ca^2+^]_cyt_) and mitochondrial Ca^2+^ ([Ca^2+^]_mito_) were measured using Fura-2AM and Rhod-2AM (Thermo Fisher Scientific, Waltham, MA) as previously described [20]. Briefly, for [Ca^2+^]_cyt_, fluorescence was measured at baseline and after treatment at an emission wavelength of 510 nm and excitation wavelengths of 340 and 380 nm. The data are presented as the fluorescence ratio of the excitation at 340 and 380 nm to the emission at 510 nm. For [Ca^2+^]_mito_, fluorescence was measured at an emission wavelength of 581 nm and an excitation wavelength of 552 nm at baseline and after treatment. The data are presented as *F*/*F*_0_, where *F* is the emission at 581 nm induced by excitation at 552 nm and *F*_0_ is the value during the pretreatment period in each experiment.

### 2.4. Measurement of Mitochondrial Respiratory Function

Mitochondrial respiratory function was determined in a 2-channel titration injection respirometer with a coupled fluorospectrometer (Oxygraph-2k; Oroboros Instruments, Innsbruck, Austria). Human-cultured fibroblasts were pretreated with TGF*β*1 (10 ng/mL) for the indicated time or kept as controls. The cells were resuspended in mitochondrial respiration medium (MiR05) for high-resolution respirometry. DatLab software 6.1 (Oroboros Instruments, Innsbruck, Austria) was used [19].

### 2.5. Measurement of ROS Levels

ROS levels were measured with a dihydroethidium (DHE) fluorescent probe for cytosolic ROS detection or with MitoSOX Red (Thermo Fisher Scientific, Waltham, MA) for mitochondrial ROS detection using a Fluoroskan Ascent Fluorometer (Thermo Fisher, Helsinki, Finland) [20].

### 2.6. Immunohistochemistry and Immunofluorescence

Tissue sections were blocked and incubated with an anti-TRPC3 antibody (1 : 100, Alomone Labs, Jerusalem, Israel) and an anti-TGF*β*1 antibody (1 : 100, Abcam, Cambridge, UK) for 2 h at room temperature. The sections were incubated with a biotinylated secondary antibody for 30 minutes and developed with ABC complex (VECTASTAIN ABC System, Vector Labs, CA, USA). Immunofluorescence was performed with an anti-*α*SMA antibody (1 : 100, Abcam, Cambridge, UK) followed by an Alexa Fluor 488-labelled secondary antibody (Abcam, Cambridge, UK). Nuclei were identified by DAPI staining. To quantify fluorescence, the glass slides were examined under an inverted fluorescence microscope (Nikon TE2000-U; Olympus, Tokyo, Japan). The percentages of myofibroblast cells (*α*SMA^+^, green) among total cells were quantified using NIS-Elements 3.0 software (Nikon Instruments).

### 2.7. Reagents and Western Blot Analysis

Human TGF*β*1 (Solarbio, No. P00121) was suspended as recommended by the manufacturer, at 10 *μ*g/mL stock concentration. Aliquots were kept frozen at −20°C until used. TRPC3 specific inhibitor Pyr3 was purchased from Sigma-Aldrich (St. Louis, MO).

Western blot assays were conducted as previously described [19, 20]. The primary antibodies included anti-TRPC3 from Alomone Labs (Jerusalem, Israel); anti-TGF*β*1 and anti-*α*SMA from Abcam (Cambridge, UK); anti-fibronectin, anti-NOX4, anti-phosphorylated Smad2/3 (pSmad2/3), anti-Smad2/3, and anti-GAPDH from Santa Cruz Biotechnology (Dallas, TX); anti-Col1a1 from Cell Signaling Technology; and antiphosphorylated pyruvate dehydrogenase E1a subunit (PDHE1a) (p-PDHE1*α*) from Merck-Millipore (Darmstadt, Germany).

### 2.8. Real-Time Quantitative Reverse Transcriptase-Polymerase Chain Reaction (RT-PCR)

Total RNA was extracted from mononuclear cells using TRIzol reagent (Invitrogen), and first-strand cDNA was synthesized with Evoscript Universal cDNA Master. One microlitre of 1 : 5-diluted first-strand cDNA was added to each 20 *μ*L PCR system and amplified with a FastStart Essential DNA Green Master RT-PCR kit. The amplification process was divided into three steps according to the manufacturer-recommended settings of the LightCycler 96 (Roche). The fluorescence reaction curves were analysed with LightCycler 96 software (version 1.1). GAPDH was used as the internal reference gene in the experiment. The primer sequences for the target gene TRPC3 (accession number NM_003305) were CAAGAATGACTATCGGAAGC (forward) and GCCACAAACTTTTTGACTTC (reverse), and those for GAPDH (accession number NM_002046) were AACTGCTTAGCACCCCTGGC (forward) and ATGACCTTGCCCACAGCCTT (reverse). The expected amplicon sizes were 203 bp (TRPC3) and 202 bp (GAPDH).

### 2.9. Statistical Analysis

The data are presented as the mean ± SEM. Unpaired Student's *t*-test was used to analyse differences between two groups. All statistical analyses were performed using SPSS software version 22.0 (IBM, Armonk, USA) and GraphPad Prism software version 6.0 (GraphPad Software, CA). *p* values less than 0.05 were considered to indicate statistical significance.

## 3. Results

### 3.1. Increased TRPC3 Promoted Fibroblast Transdifferentiation into Myofibroblasts

**Figure 1:**
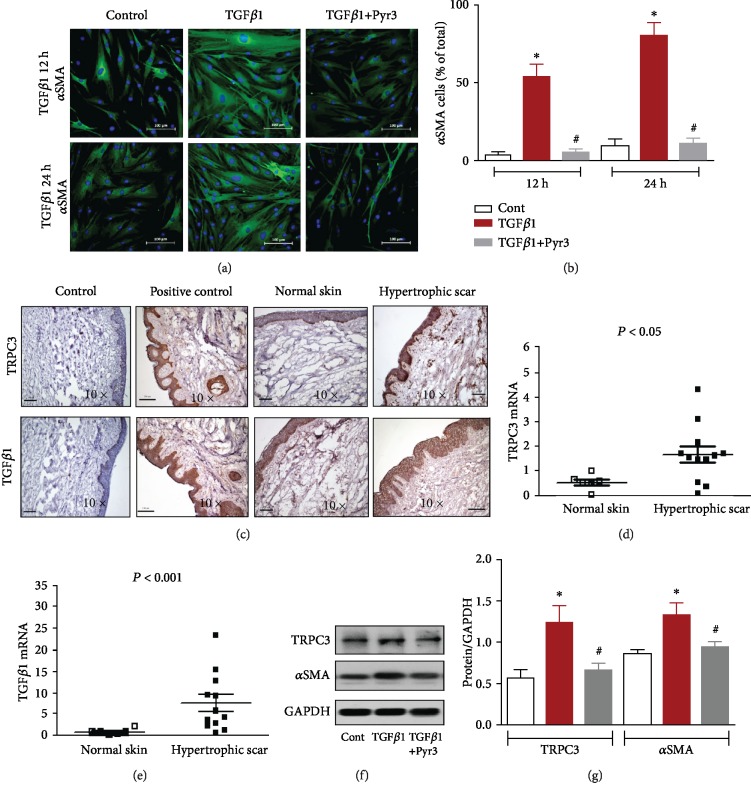
Effects of TRPC3 on myofibroblast transdifferentiation of human fibroblasts. Human fibroblasts were treated with TGF*β*1 alone (10 ng/mL) or with TGF*β*1 and Pyr3 (10 *μ*mol/L) for 12 h (upper panel) or 24 h (lower panel). (a) Immunofluorescence staining and (b) myofibroblasts (*α*SMA^+^, green) and nucleic acid staining (blue). This experiment was repeated three times, and the percentage of *α*SMA^+^ myofibroblasts was quantified over the three experiments. The bars are 100 *μ*m. ^∗^*p* < 0.05, ^#^*p* > 0.05 vs. control (cont); *n* = 3 independent experiments. (c) Immunohistochemical staining of TRPC3 and TGF*β*1 expression in hypertrophic scar (HTS) tissues and normal skin tissues. Vascular smooth muscle cells were stained with anti-TRPC3 and anti-TGF*β*1 antibodies in HTS tissue sections as internal positive controls. The scale bar represents 100 *μ*m in the 10x images. (d, e) TRPC3 and TGF*β*1 mRNA levels as assessed by RT-PCR in human HTS tissues and normal skin tissues. *p* < 0.05, *p* < 0.001 vs. normal skin tissues by Student's *t*-test. (f, g) Western blot analysis of TRPC3 and *α*SMA in human fibroblasts under treatment with TGF*β*1 alone (TGF*β*1, 10 ng/mL) or with TGF*β*1 and Pyr3 (10 *μ*mol/L) for 24 h or under control conditions without TGF*β*1 treatment. ^∗^*p* < 0.05, ^#^*p* > 0.05 vs. control (cont).

First, we investigated the effect of TRPC3 on myofibroblast transdifferentiation by immunofluorescence staining. TGF*β*1 treatment time-dependently increased myofibroblast transdifferentiation by increasing the expression of the myofibroblast marker *α*SMA in cultured human fibroblasts. In contrast, treatment with the TRPC3 inhibitor Pyr3 significantly reduced *α*SMA expression in cultured human fibroblasts compared with that in control cells (Figures 1(a) and 1(b)). Immunohistochemical staining showed that TRPC3 and TGF*β*1 more strongly expressed the epidermis and dermis in human HTS tissues than in normal skin tissues (Figure 1(c)). RT-PCR showed that TRPC3 mRNA (Figure 1(d)) and TGF*β*1 mRNA levels (Figure 1(e)) were higher in human HTS tissues than in normal skin tissues. TGF*β*1 treatment elevated TRPC3 and *α*SMA protein expression, but administration of Pyr3 significantly reduced TRPC3 and *α*SMA expression in cultured human fibroblasts (Figures 1(f) and 1(g)). These data indicate that TRPC3 is involved in human hypertrophic scarring and that inhibition of TRPC3 decreases TGF*β*1-induced myofibroblast transdifferentiation.

### 3.2. Inhibition of TRPC3 Attenuated TGF*β*1-Induced Mitochondrial Ca^2+^ Homeostasis in Human Fibroblasts

**Figure 2:**
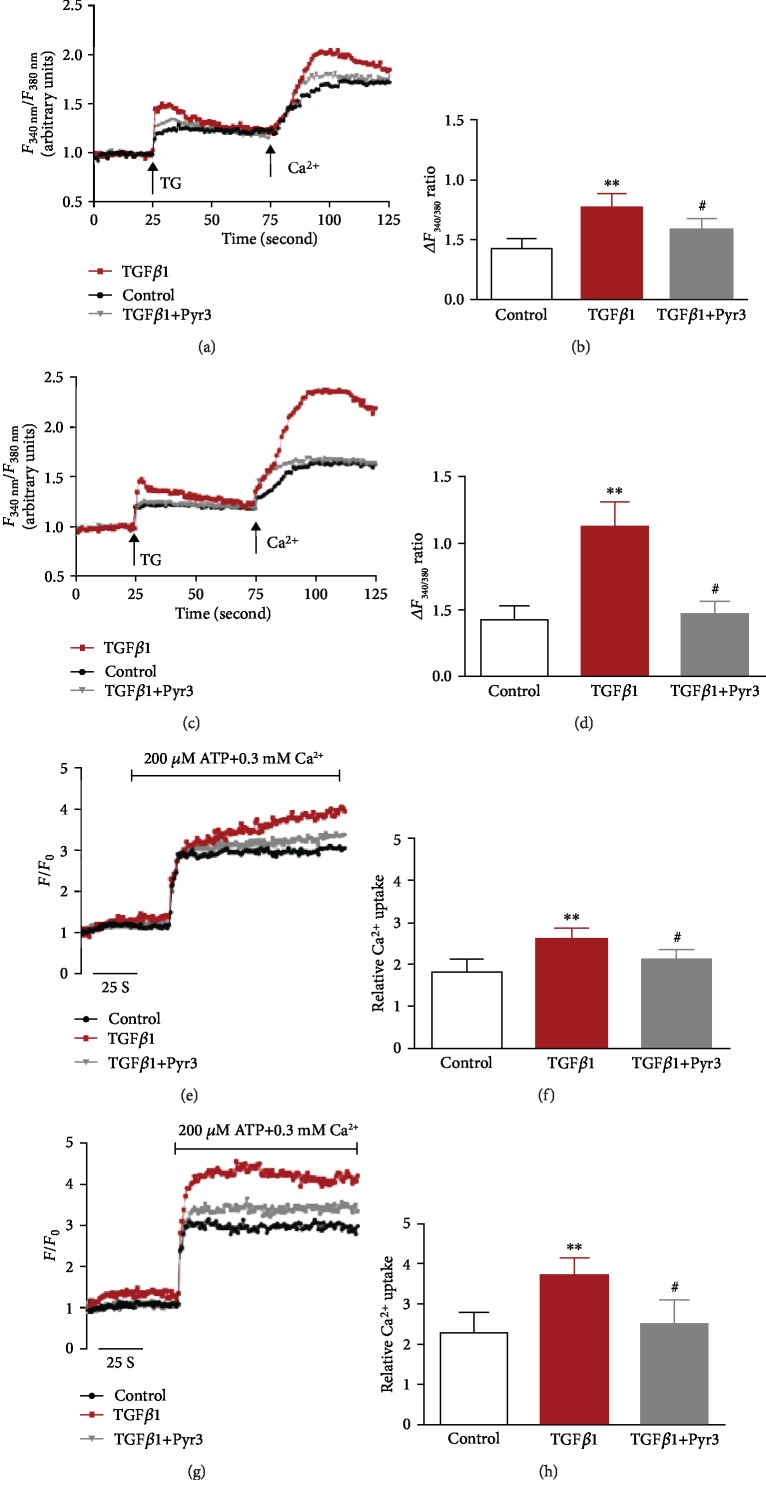
Effect of TRPC3 inhibition on cytoplasmic and mitochondrial Ca^2+^ homeostasis in human fibroblasts. SOCE was measured with thapsigargin (TG), and (a–d) [Ca^2+^]_cyt_ and (e–h) [Ca^2+^]_mito_ were quantified in human primary-cultured fibroblasts under control conditions or under TGF*β*1 (10 ng/mL) treatment for 12 h (a, b, e, f) or 24 h (c, d, g, h). TG (1 *μ*mol/L) was added after preincubation with Pyr3 (10 *μ*mol/L). The ATP concentration was 200 *μ*mol/L in a 0.3 mmol/L extracellular Ca^2+^ solution after preincubation with Pyr3 (10 *μ*mol/L). *n* = 6 per group. ^∗∗^*p* < 0.01, ^#^*p* > 0.05 vs. controls.

Next, to assess the effects of the TGF*β*1-stimulated increases in TRPC3, we examined the changes in cytosolic and mitochondrial Ca^2+^ handling in human fibroblasts. Administration of TGF*β*1 time-dependently elevated [Ca^2+^]_cyt_ (Figures 2(a)–2(d)) and [Ca^2+^]_mito_ (Figures 2(a)–2(h)) in human fibroblasts. In contrast, administration of the TRPC3 inhibitor Pyr3 significantly attenuated the effects of TGF*β*1. These results suggest that TGF*β*1-mediated enhancement of TRPC3 function contributes to regulating mitochondrial Ca^2+^ handling in human fibroblasts.

### 3.3. Inhibition of TRPC3 Reduced ROS Production and Improved Mitochondrial Function in Human Fibroblasts

**Figure 3:**
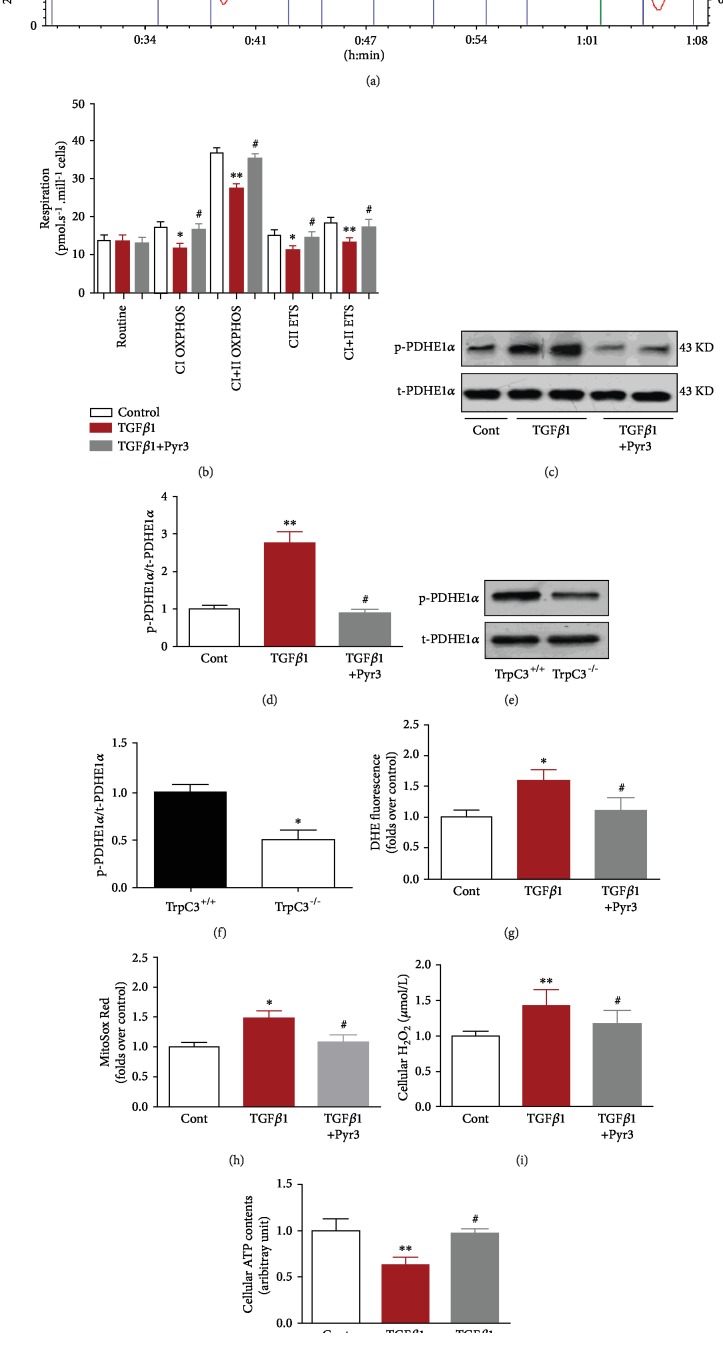
Effects of TRPC3 on ROS production and mitochondrial function in human fibroblasts. Mitochondrial oxygen consumption was measured in human fibroblasts treated with TGF*β*1 (10 ng/mL) for 24 h via Oxygraph-2k high-resolution respirometry. (a) The test protocol is shown. (b) The values are expressed in pmol/s per 10^6^ cells. Summarized data for oxygen consumption capacity. *N* = 6, ^∗^*p* < 0.05, ^∗∗^*p* < 0.01, ^#^*p* > 0.05 vs. controls. (c, d) p-PDHE1*α* and total PDHE1*α* (t-PDHE1*α*) protein expression in human fibroblasts treated with TGF*β*1 alone (10 ng/mL) or with TGF*β*1 and Pyr3 (10 *μ*mol/L) for 24 h. ^∗∗^*p* < 0.01, ^#^*p* > 0.05 vs. controls. (e, f) p-PDHE1*α* and t-PDHE1*α* protein expression in dermal fibroblasts from WT or *Trpc3^−/−^* mice. ^∗^*p* < 0.05 vs. WT. (g) DHE staining for cellular ROS, (h) mitochondrial ROS levels, (i) H_2_O_2_ production, and (j) ATP levels in human fibroblasts under TGF*β*1 treatment (10 ng/mL) or TGF*β*1 and Pyr3 treatment (10 *μ*mol/L) for 24 h. *N* = 6, ^∗^*p* < 0.05, ^∗∗^*p* < 0.01, ^#^*p* > 0.05 vs. controls.

We then investigated the effects of TGF*β*1 and Pyr3 treatment on mitochondrial ROS production and respiratory functions in human fibroblasts. Specifically, compared with the control treatment, TGF*β*1 treatment significantly reduced the values of mitochondrial respiratory function parameters, such as CIOXPHOS, CI+IIOXPHOS, CIIETS, and CI+IIETS, in human fibroblasts. However, Pyr3 treatment significantly reversed the changes in these mitochondrial parameters (Figures 3(a) and 3(b)). p-PDHE1*α* levels were significantly increased in TGF*β*1-treated human fibroblasts, but total PDHE1a levels were not altered (Figures 3(c) and 3(d)). The wound granulation tissues from *Trpc3^−/−^* mice had lower p-PDHE1*α* levels than those from *Trpc3^+/+^* mice (Figures 3(e) and 3(f)). Furthermore, administration of TGF*β*1 increased cellular and mitochondrial ROS and H_2_O_2_ production and reduced ATP levels in human fibroblasts compared with those in control cells, but Pyr3 treatment significantly reversed these changes (Figures 3(g)–3(j)). These results indicate that inhibition of TRPC3 reduces TGF*β*1-induced ROS production and improves mitochondrial function in human fibroblasts.

### 3.4. TRPC3 Deficiency Attenuated Myofibroblast Transdifferentiation by Inhibiting the NOX4/pSmad Pathway

**Figure 4:**
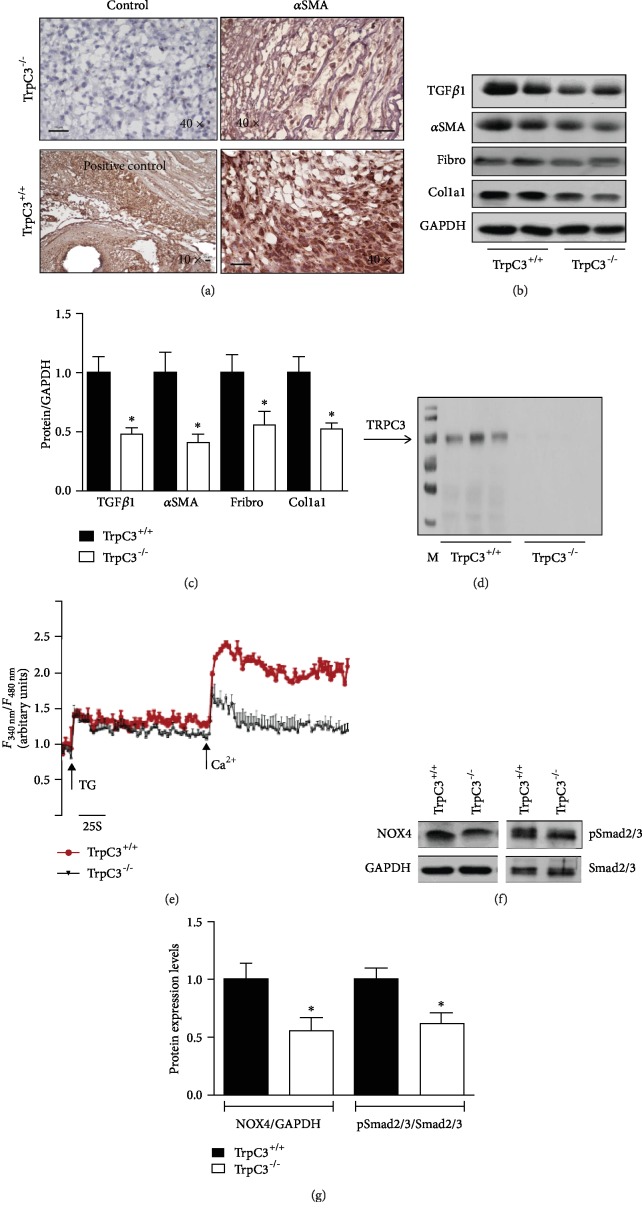
*Trpc3^−/−^* attenuated myofibroblast transdifferentiation through inhibition of NOX4/pSmad in vivo. Wound granulation tissues were harvested 6 days after wounding, and immunochemistry with a primary antibody against *α*SMA was performed. (a) Representative immunohistochemical images showing *α*SMA expression in wound granulation tissues from *Trpc3^+/+^* mice and *Trpc3^−/−^* mice. Vascular smooth muscle cells were stained with an anti-*α*SMA antibody in granulation tissues from *Trpc3^+/+^* mice as an internal positive control. The scale bar represents 100 *μ*m. (b, c) Western blot analysis of TGF*β*1, *α*SMA, fibronectin (Fibro), and Col1a1 levels in wound granulation tissues from *Trpc3^+/+^* and *Trpc3^−/−^* mice. *n* = 6. ^∗^*p* < 0.05 vs. *Trpc3^+/+^* mice. (d) TRPC3 immunoreactivity was detected with an anti-TRPC3 antibody (96 kDa) in homogenates of primary fibroblasts from *Trpc3^+/+^* mice but not in those from *Trpc3^−/−^* mice. (e) Quantification of thapsigargin (TG, 1 *μ*mol/L)-induced SOCE and additional Ca^2+^ (1 mmol/L) in dermal fibroblasts from *Trpc3^+/+^* and *Trpc3^−/−^*mice. (f, g) Western blot analysis of NOX4, pSmad2/3, and Smad2/3 in wound granulation tissues from *Trpc3^+/+^* and *Trpc3^−/−^* mice. *n* = 3. ^∗^*p* < 0.05 vs. *Trpc3^+/+^* mice.

We further investigated the effect of TRPC3-mediated Ca^2+^ signalling on myofibroblast transdifferentiation in vivo. 8 mm wounds were created in the skin on the backs of *Trpc3^+/+^* and *Trpc3^−/−^* mice. The wound granulation tissues were harvested after 6 days and assessed by immunochemical staining with an anti-*α*SMA antibody. The myofibroblast marker *α*SMA was abundantly expressed in the wound granulation tissues from *Trpc3^+/+^* mice compared with those from *Trpc3^−/−^* mice (Figure 4(a)). TRPC3 deficiency significantly decreased *α*SMA, TGF*β*1, fibronectin, and Col1a1 protein expression in wound granulation tissues (Figures 4(b) and 4(c)). Furthermore, western blotting confirmed that the TRPC3 protein was expressed in dermal fibroblasts from *Trpc3^+/+^* mice but not in those from *Trpc3^−/−^* mice (Figure 4(d)). *Trpc3^−/−^* mice exhibited significantly lower levels of store-operated calcium entry (SOCE) in dermal fibroblasts after TGF*β*1 treatment than *Trpc3^+/+^* mice (Figure 4(e)). Taken together, these results suggest that TRPC3 deficiency attenuates myofibroblast transdifferentiation by inhibiting the NOX4/pSmad2/3 pathway during wound healing (Figures 4(f) and 4(g)).

## 4. Discussion

**Figure 5:**
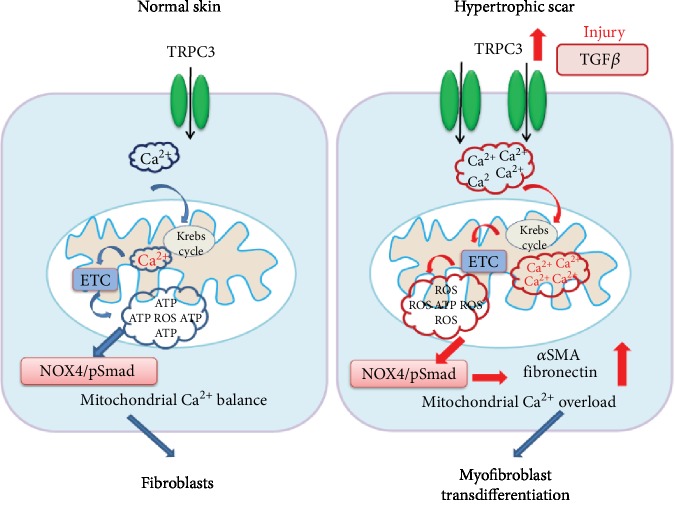
Schematic illustration depicting the mechanism by which enhanced TRPC3-mediated mitochondrial Ca^2+^ homeostasis and ROS generation contribute to myofibroblast transdifferentiation via the NOX4/pSmad pathway in the hypertrophic scars.

Hypertrophic scar (HTS) is a devastating sequela of injury characterized by overproliferation of *α*SMA-expressing myofibroblasts. Here, we verified that TRPC3 participates in abnormal mitochondrial Ca^2+^ homeostasis and ROS production and promotes *α*SMA myofibroblast differentiation during wound healing. TRPC3 and TGF*β*1 mRNA expression levels were increased in fibroblasts from HTS tissues. TRPC3 deficiency decreased TGF*β*1-induced SOCE-mediated Ca^2+^ influx and *α*SMA, TGF*β*1, fibronectin, and Col1a1 protein expression in fibroblasts or wound granulation tissues. TRPC3 deficiency attenuated hypertrophic scar through inhibition of the NOX4/pSmad2/3 pathway in vivo (Figure 5). Our findings highlight essential roles for TRPC3-mediated mitochondrial Ca^2+^ handling and ROS production in regulating myofibroblast transdifferentiation during wound healing.

TRP channels are associated with wound healing in different tissue types [21–24]. TRPV2 inhibitor has been found to attenuate fibroblast differentiation and contraction mediated by keratinocyte-derived TGF*β*1 in a rat wound healing model [21], and TRPV1-deficient mice exhibit impaired healing of corneal incision injuries [22]. TRPA1-deficient mice exhibit suppressed neovascularization of the corneal stroma [23], and activation of TRPV3-mediated Ca^2+^ influx accelerates corneal epithelial cell proliferation [24]. In addition, TRP-mediated Ca^2+^ signal transduction activates several transcription factors in the nucleus to produce cytokines and to suppress murine T-cell activation and endogenous inflammation-induced intracellular Ca^2+^ increases [25]. Furthermore, Ca^2+^ activity mediated by TRPC6 through p38 mitogen-activated protein kinase (MAPK) contributes to myofibroblast transdifferentiation [17]. In the present study, we found that TRPC3-mediated elevations in mitochondrial Ca^2+^ and ROS production regulate myofibroblast transdifferentiation during wound healing.

Mitochondrial Ca^2+^ diminishes the levels of ROS produced by complexes I and III but enhances ROS generation if these complexes are dysfunctional. Excessive mitochondrial Ca^2+^ uptake promotes ROS production [26], and oxidative stress promotes redistribution of TRPM2 to the plasma membrane in hepatocytes [27]. In addition, ROS-mediated TRPC6 activation in vascular cells is associated with abnormal vascular tone in puromycin aminonucleoside-induced podocyte injury [28]. Suppression of Nox4 decreases myofibroblast formation and fibrosis in lung, liver, kidney, and cardiac injury models [29, 30]. Furthermore, PDHE1*α* phosphorylation is mediated by redox-sensitive PDH kinase, which is activated by mitochondrial ROS [31]. However, the regulation of PDH activity during myofibroblast transdifferentiation is poorly understood. We found that TGF*β*1 enhanced PDHE1*α* phosphorylation, inhibited TRPC3 by Pyr3 reducing PDH kinase and recovering PDH activity. Recently, Ljaz T. showed that TGF*β*1-induced myofibroblast transformation is associated with the ROS-producing enzyme Nox4. Our previous study indicated that inhibition of TRPC3 attenuates mitochondrial Ca^2+^ uptake and ROS production in the vasculature of hypertensive rats. In the present study, we found that the levels of TGF*β*1-induced Ca^2+^ influx and ROS production were significantly lower in dermal fibroblasts from *Trpc3^−/−^* mice or Pyr3-treated human fibroblasts. In *Trpc3^−/−^* mice, myofibroblast transdifferentiation during wound healing was impaired due to inhibition of the Nox4/pSmad2/3 pathway.

TGF*β* signalling has been implicated in wound healing. TGF*β*1 inhibits the proliferation of keratinocytes but stimulates the proliferation of dermal fibroblasts [6]. TGF*β*1 has been found in wound fluid from chronic ulcers, and fibroblasts isolated from chronic venous ulcers show decreased levels of TGF*β* RII. Notably, disruption of the TGF*β*/Smad3 signalling pathway by loss of Smad3 confers resistance to tissue fibrosis in the skin, kidneys, lungs, and liver [9, 10]. Furthermore, Smad3 WT mice treated with 2,4,6-trinitrobenzenesulfonic acid (TNBS) develop colorectal fibrosis and exhibit upregulation of TGF*β*1, Smad3, av*β*6 integrin, and mTOR but downregulation of PPAR-*γ* [32]. PPAR-*γ* has anti-inflammatory and antifibrotic effects in inflammatory bowel disease related to platelet-derived growth factor (PDGF), IL-1, and TGF*β* [33]. Activation of TGF*β*1/Smads by mast cell chymase promotes HTS fibroblast proliferation and collagen synthesis [34]. In the present study, our data demonstrated that inhibition of TRPC3 attenuated TGF*β*1-induced myofibroblast transdifferentiation by inhibiting ROS production and alleviated abnormalities in mitochondrial respiratory function in fibroblasts. These findings suggest that TRPC3 upregulation-mediated ROS activation of the Nox4/pSmad2/3 pathway may be the mechanism by which TGF*β*1 promotes myofibroblast transdifferentiation during wound healing.

In summary, we tested the hypothesis that TGF*β*1 activates TRPC3-mediated mitochondrial Ca^2+^ homeostasis and ROS production to promote myofibroblast transdifferentiation and HTS formation.

## 5. Conclusions

The results of this study demonstrate a potential mechanism of TGF*β*1-enhanced TRPC3 activity at the cytoplasmic and mitochondrial levels, which contributes to mitochondrial dysfunction in dermal fibroblasts after injury. TRPC3 can regulate [Ca^2+^]_mito_, ROS production, and mitochondrial energy metabolism to promote myofibroblast formation during wound healing.

## Data Availability

The materials in this manuscript are available from the corresponding author on reasonable request.
